# Severe Hepatitis Outbreak Post COVID-19: How Do We Protect Our Children?

**DOI:** 10.7759/cureus.28793

**Published:** 2022-09-05

**Authors:** Zahra Abbas, Omer A Shaikh, Manisha Essarani, Rameel M Aftab, Abdulqadir J Nashwan

**Affiliations:** 1 Department of Medicine, Dow University of Health Sciences, Karachi, PAK; 2 Department of Internal Medicine, Ziauddin University, Karachi, PAK; 3 Department of Medicine, Ziauddin University, Karachi, PAK; 4 Nursing Education, Hamad Medical Corporation, Doha, QAT

**Keywords:** outbreak, children, pediatrics, covid-19, hepatitis

## Abstract

This editorial highlights the alarming increase in pediatric cases of severe acute hepatitis of unknown etiology. Global strategies should be implemented to prevent the current high workload of the healthcare system from getting worse.

## Editorial

There has been an alarming increase in cases of severe acute hepatitis of unknown etiology since April 5^th^, 2022, until which only 10 cases had been reported, mainly from central Scotland, and within the next three days, upon further investigation, 74 cases had been discovered across the United Kingdom in previously healthy young children [1]. Consequently, World Health Organization (WHO) declared the outbreak on June 24^th^. Not only are the more common Hepatitis A, B, C, and D viruses not the ones that cause the disease, but the disease has mostly affected children under the age of six, who usually do not get severe hepatitis. In addition, studies that are still ongoing have shown that Adenovirus and Severe Acute Respiratory Syndrome coronavirus 2 (SARS Cov2) may play a role in how the disease develops.

Since April 5^th^, 1010 cases, 22 deaths, and 46 liver transplants have been recorded by 35 countries, with about half the cases from Europe, 33% from the region of the Americas, 7% in the Western Pacific, and 2% in Southeast Asia. However, it's important to note that the lower number in regions other than the Americas and Europe may be justified by the lack of advanced surveillance and reporting systems. Cases have continued to rise, and three additional countries have reported further cases, which await classification. According to PCR studies, Adenovirus, which normally only causes flu-like symptoms, was discovered in 52% of cases in Europe and 9% in Japan. While SARS-Cov-2 was found in 16% of cases in Europe and 8% in the USA and Japan [2], it is unclear which of the two viruses is directly responsible for the disease and whether they work together. Some theories suggest Adenovirus may damage the liver directly, while others state that it might result from a hyperactive and prolonged immune response following SARS-CoV-2 infection. This information is important because the first would need antiviral treatment, and the second would need drugs that weaken the immune system. If the inflammation is caused by Adenovirus directly, this could have serious consequences [3].

The WHO therefore stresses collaborative efforts between affected countries, who should promptly report cases using WHO’s clinical case report form, collect multiple specimens as early as possible, considering these viruses remain in the blood for a limited amount of time, and ensure their proper storage, or in the case of lack of storage facilities, use regional or global laboratories. Patients who show signs of severe hepatitis and have risk factors for COVID-19 must be isolated and tested, especially since the disease may be epidemiologically linked. In addition, general hygiene rules must be followed, like washing hands well, avoiding sick crowds, and storing food properly [2].

Only recently were the COVID-19 vaccines developed and approved by the Food and Drug Administration (FDA) for children under five years of age [4]. Since this is the age group most affected by severe hepatitis and their blood samples show the presence of COVID-19 and Adenovirus, vaccine administration must be conducted as quickly as possible. Furthermore, following the relaxation of COVID-19 restrictions in the UK, hepatitis C cases rose considerably, which points to the possibility of exposure to a myriad of viruses, such as coronavirus or Adenovirus, all at once rather than gradually, which might have been what caused the steep increase in cases. Keeping all these factors in mind, quarantine restrictions, especially the closure of schools and commencement of online classes, should be tightened, at least until the vaccination of children in all those areas facing the outbreak of hepatitis is completed. By vaccinating all children worldwide, the disease will not spread, especially in third-world countries with few medical resources post-COVID-19.

The aforementioned strategies will ensure that the pre-existing burden on the healthcare system does not worsen further because we would then have to handle COVID-19 and its possible secondary effects, such as hepatitis and liver transplants. Furthermore, local governments should engage in hepatitis virus epidemic investigations by conducting regular and cutting-edge serologic and molecular techniques on clinical samples, which will enhance the early detection of cases and improve the surveillance system.
